# Lattice Constant Effect on Diffracted Transmission of ITO Periodic Nanostructures and Improvement of the Light Extraction Efficiency of Light-Emitting Diodes

**DOI:** 10.3390/mi12060693

**Published:** 2021-06-14

**Authors:** Zhanxu Chen, Runhong Ding, Feng Wu, Wei Wan

**Affiliations:** School of Optoelectronic Engineering, Guangdong Polytechnic Normal University, Guangzhou 510665, China; ding_rh@163.com (R.D.); fengwu@gpnu.edu.cn (F.W.); wanwei@gpnu.edu.cn (W.W.)

**Keywords:** light-emitting diodes (LEDs), diffraction, nanostructures

## Abstract

We studied the effects of the lattice pitch of indium-doped tin oxide (ITO) periodic nanostructures on the diffracted transmission to improve the light extraction efficiency of light-emitting diodes (LEDs). Periodic hexagonal ITO nanopillars with lattice constants of 600, 800, 1050, 1200, and 1600 nm were fabricated on ITO electrodes. We found that the light extraction efficiency strongly depended on the lattice constant. The LEDs with a lattice constant of 800 nm ITO nanopillars showed an increase in light extraction of 83%. In addition, their electrical properties were not degraded compared to conventional LEDs. The dependence of the extraction efficiency on the lattice constant was also calculated using a 3D finite-difference time-domain (FDTD) method, and this dependence was in good agreement with the experimental measurements. The transmission of each diffraction order and with the total transmission of ITO nanopillars with different lattice constants were calculated using the FDTD method to investigate the enhancement effect.

## 1. Introduction

To date, light emitting diodes (LEDs) have been widely utilized as energy-saving and environment-friendly light sources [1,2,3,4,5]. Research is ongoing to improve LED performance through more efficient light-generating and light-extracting structures [6,7]. However, owing to the large difference in the refractive indices between the semiconductor materials and the air, conventional LEDs still suffer from limited light extraction [8,9,10,11]. In the past three decades, a kind of typical periodic micro-structures—photonic crystals (PhCs)—have attracted great interest due to their potential applications in the design of optical reflectors [12,13], absorbers [14], nanosieves [15], and filters [16,17]. 

Researchers have incorporated PhCs into LEDs and greatly improved the light extraction efficiency [6,7,8,11,18,19,20,21,22]. In most publications, the PhCs act as diffraction gratings to extract guided light. The light extraction enhancement factor is affected by the structural parameters, such as the lattice constant, filling factor, and etch depth. Among these, the lattice constant plays an important role in light extraction enhancement. In particular, Kim et al. made the air-hole array patterns with a period that varied from 300 to 700 nm to investigate PhC GaN-based LEDs with peak wavelength of *λ* = 400 nm and found the best enhancement factor was 2.1 when the lattice constant was 500 nm [22]. 

Shin et al. investigated the enhancement of light extraction in GaN LEDs with a variety of PhC structures on either the indium-tin-oxide (ITO) or p-GaN layers [23]. Kim et al. fabricated PhC slabs with triangular lattice constants of 230, 345, 460, and 690 nm onto the p-GaN surfaces and proved that the highest enhancement factor of light output was 3.5 when the lattice constant was 460 nm [24]. Xiong et al. studied the light extraction effects of the lattice pitch of square-lattice PhCs on the diffracted transmission [25,26]. Therefore, the investigation of the light extraction effects and the physical mechanism of nanostructures with different lattice constants are a hot topic.

In this paper, we experimentally and theoretically study the optical output enhancement in GaN LEDs with a variety of hexagonal nanopillar array structures with different lattice constants on the ITO layer. The experimental results are confirmed by three-dimensional (3D) finite difference time-domain (FDTD) simulations. The physical mechanism of light extraction based on diffraction theory is discussed in detail.

## 2. Experimental Methods

The entire LED epitaxial wafer consists of a 2-inch sapphire (Al_2_O_3_) substrate, a 2-µm undoped GaN (u-GaN) buffer layer, a 3-µm n-GaN layer, an active layer of five-period InGaN/GaN MQWs, and a 150-nm p-GaN layer. A transparent ITO electrode with a thickness of about 360 nm was first deposited on the p-GaN surface. The LED chips with dimensions of 300 × 300 μm were formed by mesa-etching the exposed n-type GaN via standard lithography, ITO wet etching, and subsequent inductively coupled plasma (ICP) etching. Cr/Pt/Au was deposited on the top of ITO surface as well as the exposed n-GaN layer as a contact metal for both the p- and the n-GaN layers. 

The ITO nanopillar array was fabricated using the nanosphere lithography method [27]. To study the influence of lattice constants on the light extraction efficiency (LEE) of GaN-based LEDs, we fabricated a nanopillar array with various lattice constants on the ITO surface of the LEDs. All of the devices were fabricated from the same LED wafer in order to eliminate the variations from wafer to wafer and to ensure the reliability of the results. The microscopic electroluminescence (EL) was measured by employing a system assembled with an optical microscope, an I-V characteristic instrument, and an integrating sphere.

## 3. Results and Discussion

**Figure 1:**
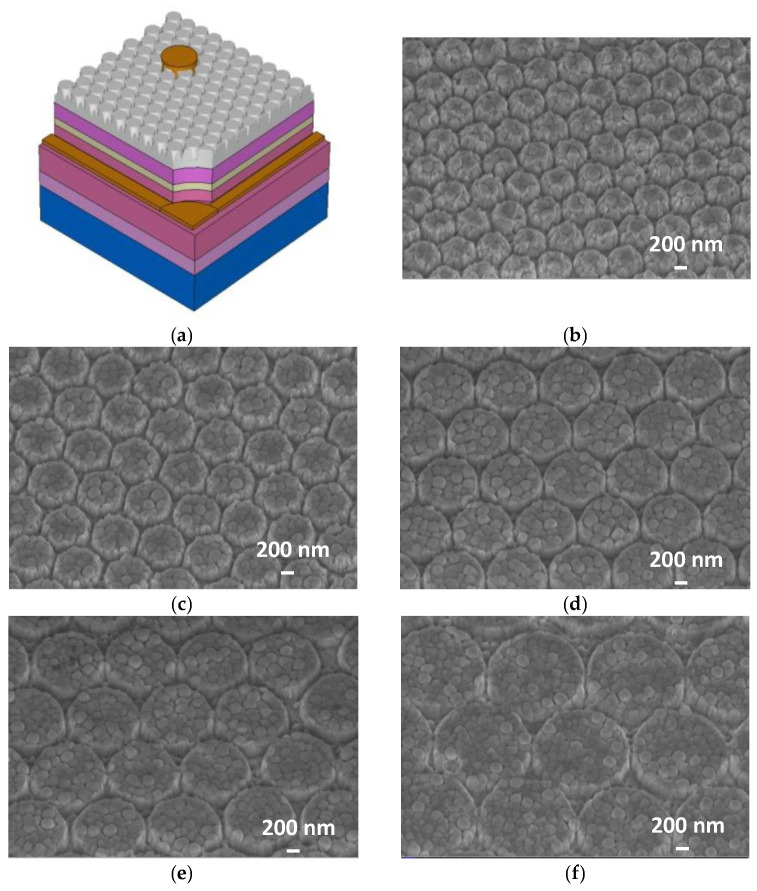
(**a**) Schematic illustration of the LED with ITO nanopillar array. (**b**–**f**) 30 degree tilt SEM images of the ITO nanopillar of samples (**b**) B, (**c**) C, (**d**) D, (**e**) E, and (**f**) F.

In the experiments, a 2-inch LED wafer was divided into six parts to prepare the samples. Sample A was a conventional LED, which acted as a reference device. Samples B, C, D, E, and F were fabricated nanopillar arrays with various lattice constants of about 600, 800, 1000, 1200, and 1600 nm, respectively. Figure 1a presents the schematic of the complete structure of GaN-based LEDs with periodic nanopillar arrays on ITO layer, and Figure 1b–f, respectively, shows the ITO nanopillar array morphologies in samples B, C, D, E, and F.

**Figure 2:**
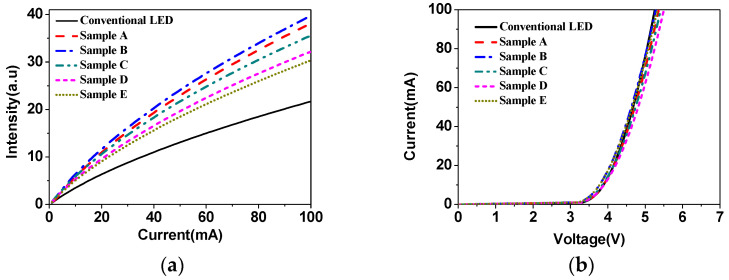
Electroluminescence curves of five nanopillar patterned LEDs and the conventional LED. (**a**) The light output intensity versus injection current (L-I) characteristics. (**b**) Current versus voltage (I-V).

Figure 2a illustrates the injection current-light output (I-L) characteristics from various samples with different lattice constants of nanopillar arrays on ITO layer. Compared to the conventional LED sample A, the light output power (LOP) of samples B, C, D, E, and F are enhanced by 1.76, 1.83, 1.64, 1.48, and 1.4 times, respectively, at an injection current of 100 mA. From the above results, the extraction efficiency was seen to be the largest when the lattice constant is 800 nm. Figure 2b shows the injection current–voltage (I-V) characteristics of the LEDs with and without a nanopillar array on the ITO layer measured at room temperature. It is clear that the LED with nanopillar array exhibited nearly the same I-V characteristics as the conventional LED.

To verify the experiments, FDTD simulations were carried out to investigate the lattice constants effect of the periodic nanopillar arrays on the light extraction of the LEDs. The simulated LED structure consisted of an infinite sapphire substrate, a 5200-nm GaN layer (including an n-GaN layer, a MQW, and a p-GaN layer), and a 360-nm ITO layer. In the simulation, multiple dipoles were used as point light sources. The wavelength of incident light was set to be 453 nm, which corresponds to the center wavelength of the emission spectrum. 

**Figure 3:**
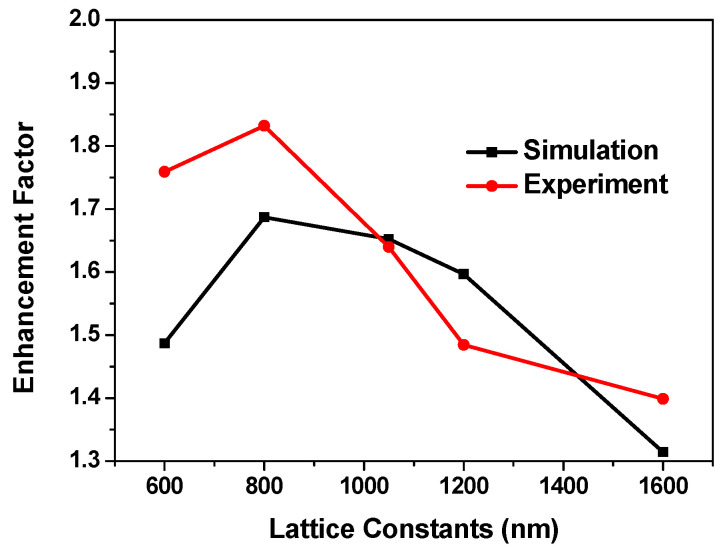
Dependence of the experimental and calculated enhancement factors of light extraction on the lattice constant.

Nanopillar arrays with various lattice constants between 600 and 1600 nm were modeled. In the simulations, perfectly matched layers (PML) absorbing boundary conditions were assumed on all sides of the model. There were about 20 × 20 nanospheres in the simulation region. Figure 3 shows the calculated and experimental light-extraction enhancement factors of LEDs as a function of lattice constants. The trend of the calculated results was consistent with the experimental ones. The experimental and calculated results showed that optimum extraction efficiency was achieved when the lattice constant was about 800 nm. 

As shown in Figure 3, some obtained values were different between the experimental and the simulated results. It is difficult to give a reason for such differences; however, we propose that it is partly due to the differences of the morphology of ITO nanopillar between the experimental and the simulated settings. Further investigation is required to clarify this.

**Figure 4:**
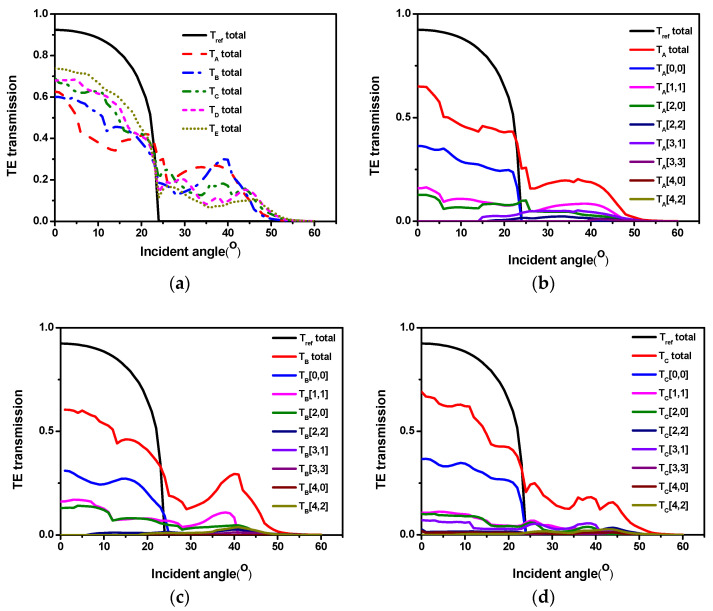

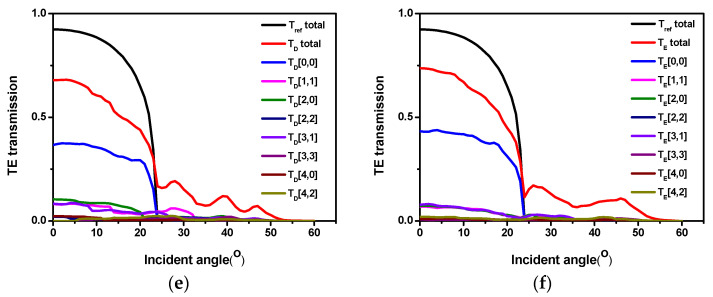
(**a**) Simulated relationships between the transmission and incident angle at a wavelength of 453 nm for LEDs with and without ITO periodic nanopillar arrays. The transmission of the total and each diffraction order as a function of the incident angle for nanopillars with different lattice constants: (**b**) 600 nm, (**c**) 800 nm, (**d**) 1050 nm, (**e**) 1200 nm, and (**f**) 1600 nm.

In most LEDs operating in the visible and near-infrared wavelength ranges, TE mode emission is dominant [28]. To investigate the physical mechanism of the LEE enhancement, the TE transmission angular spectra of the related structures at the emission wavelength of 453 nm were simulated by the FDTD method, as shown in Figure 4a. For the reference LED, the transmission dropped to zero beyond the critical angle of about 23.7 degrees due to the total internal reflection. However, for the LEDs with ITO periodic nanopillar arrays, there was still extra transmission beyond the critical angle. This feature implies that the incident light beyond the critical angle can be partially extracted, leading to an enhanced extraction efficiency. This indicates that light with incident angles of less than 60 degrees can be leaky. 

Additionally, the transmission of the LEDs with ITO periodic nanopillar arrays is lower than that of the reference LED below the critical angle, which means that some of the original extracted modes are coupled to the reciprocal lattice and turn back as trapped guided modes [25,26,29]. The transmission increased gradually below the critical angle when the lattice constant of periodic nanopillar arrays changed from 600 to 1600 nm. However, beyond the critical angle, the transmission decreased gradually when the lattice constant of periodic nanopillar arrays changed from 600 to 1600 nm. Therefore, there should be an optimal period with high transmission in a wide range of incident angles.

In order to better understand the transmission angular spectrum, we calculated the diffraction angular spectrum corresponding to the transmission angular spectrum with difference lattice constants. The relations of total transmission of various samples with incident angles are shown in Figure 4a. Figure 4b–f presents the typical transmission profiles of each diffraction order together with total transmission of the periodic nanopillar arrays as a function of the incident angle for the nanopillars with lattice constants of 600, 800, 1050, 1200, and 1600 nm, respectively. 

The curves of “T_ref_ total” are the transmissions of a conventional planar LED in each figure as a reference. The curves labeled by T (m, n) represent the transmissions with the diffraction order of (±m, ±n), and the curves of “T total” are the sum of the whole diffracted transmission. The curve T (0, 0) corresponds to the direct transmission from ITO to air. In this paper, the minimum period was 600 nm, which is much larger than the critical pitch (*a*_crit_ = *λ*/(1 + *n*_eff_)); therefore, the component of higher-order diffraction appears in Figure 4b–f. The transmissions of T (0, 0) increased with the increase in the lattice constant. As a result, the total angular transmissions will be relatively high in the critical angle range. In addition, generally speaking, with the increase in the lattice constant, the diffraction order will also increase; however, we found that the diffraction energy did not increase, which meant that the relatively high period of higher-order diffraction energy was not obvious. Therefore, for a relatively good nanostructure, a relatively high transmission at all angles is preferred, and this provides a basis for us to design nanostructures.

**Figure 5:**
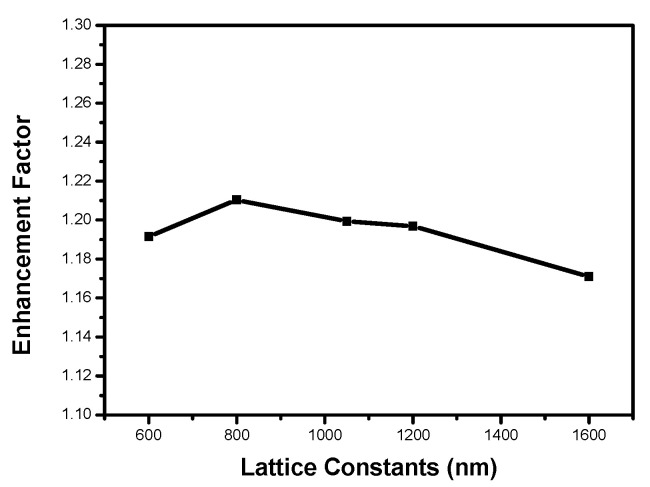
The calculated enhancement factors of the TE-polarized integrated transmission on the lattice constant.

To determine the effect of diffraction, we also calculated the integrated transmission $T=\int_{0}^{2\pi }\int_{0}^{\pi /2}T\left(\theta ,\phi \right)\sin\theta d\theta d\phi$ over the whole incident angle θ and azimuth angle φ for lattice pitches from 600 to 1600 nm for the structures in the experiments. We first integrated the transmittance of the reference sample as unity. The curve with square dots in Figure 5 shows the calculated enhancement factors of the TE-polarized integrated transmission efficiency versus the lattice pitch. The trend of the curve is basically consistent with Figure 3. The enhancement factor reached the maximum as the lattice constant was 800 nm. However, the enhancement factors were relatively small compared with those in Figure 3. In the simulation, the transmission angular spectrum did not consider the multireflection by the lower interface between GaN and sapphire substrate. The transmission below the critical angle was lower compared with the reference LED, which resulted in some extra light being diffracted back into the LED. However, the back-diffracted light can be reflected by the back of the LED and then re-extracted by the structures [30]. In addition, there was transmission beyond the critical angle. Although the transmission was not high, this indicates that the light beyond the critical angle can also increase the escape of light through multiple reflections.

The transmission integral only considers the transmission of nanostructures, and the light reflected and retransmitted many times was not included in the calculation, which leads to the result of integration being less than the experimental result. In Figure 3, multiple dipole light sources were used to simulate the light propagation in the structure, and the multireflection was also taken into account. Hence, the simulated data essentially matches with the experimental data. 

## 4. Conclusions

In summary, we fabricated a hexagonal ITO nanopillar array with lattice constants of 600, 800, 1050, 1200, and 1600 nm on LED structures. The light extraction efficiency was found to be strongly dependent on the lattice constant of the ITO nanopillar arrays. The lattice constant effect of the ITO nanopillar array on the LEE of LEDs was investigated by experiments and FDTD simulations. Compared with the corresponding conventional LEDs, the LEDs with ITO nanopillar arrays resulted in an enhancement of the LEE of 1.83-fold at a current of *I* = 100 mA. 

The results of the FDTD simulations agreed well with the experiments. The calculations of the transmissions of the total and each order of diffraction of the ITO nanopillar array demonstrated that the improvement of light extraction was achieved by the competition among the different diffraction mechanisms. Due to the different lattice constants, the high and low order diffraction transmittances showed different characteristics. This might help to understand the mechanism of light extraction enhancement from the surface diffraction structure on LEDs and provide a design basis for device applications.
